# Estimated Annual Numbers of Foodborne Pathogen–Associated Illnesses, Hospitalizations, and Deaths, France, 2008–2013

**DOI:** 10.3201/eid2309.170081

**Published:** 2017-09

**Authors:** Dieter Van Cauteren, Yann Le Strat, Cécile Sommen, Mathias Bruyand, Mathieu Tourdjman, Nathalie Jourdan Da Silva, Elisabeth Couturier, Nelly Fournet, Henriette de Valk, Jean-Claude Desenclos

**Affiliations:** Santé Publique France, Saint-Maurice, France

**Keywords:** illness, hospitalization, death, France, epidemiology, foodborne disease, surveillance, prevention, control, foodborne pathogens, bacteria, parasites, viruses

## Abstract

Estimates of the annual numbers of foodborne illnesses and associated hospitalizations and deaths are needed to set priorities for surveillance, prevention, and control strategies. The objective of this study was to determine such estimates for 2008–2013 in France. We considered 15 major foodborne pathogens (10 bacteria, 3 viruses, and 2 parasites) and estimated that each year, the pathogens accounted for 1.28–2.23 million illnesses, 16,500–20,800 hospitalizations, and 250 deaths. *Campylobacter* spp., nontyphoidal *Salmonella* spp., and norovirus accounted for >70% of all foodborne pathogen–associated illnesses and hospitalizations; nontyphoidal *Salmonella* spp. and *Listeria monocytogenes* were the main causes of foodborne pathogen–associated deaths; and hepatitis E virus appeared to be a previously unrecognized foodborne pathogen causing ≈68,000 illnesses in France every year. The substantial annual numbers of foodborne illnesses and associated hospitalizations and deaths in France highlight the need for food-safety policymakers to prioritize foodborne disease prevention and control strategies.

Foodborne pathogens are of public health concern worldwide (*1*). Estimates of the total number of foodborne illnesses and associated hospitalizations and deaths are needed to assess their effect on health and to set priorities for surveillance, prevention, and control strategies. In 2000, the number of foodborne illnesses and associated deaths in France was estimated by using data from 1990–2000. However, for most pathogens, data were lacking to derive estimates at the population level (*2*).

Since that study, specific surveillance systems have been implemented in France for *Campylobacter* spp. (2002) (*3*), hepatitis A virus (2005), and hepatitis E virus (2002) (*4*). Additional surveys have been conducted to provide information on healthcare-seeking behavior and the incidence of acute gastroenteritis in the general population (2009–2010) (*5*) and on physician practices in requesting fecal samples for patients with acute gastroenteritis (2013–2014) (*6*). Furthermore, the quality and availability of other nonspecific data sources (e.g., hospital discharge registers and health insurance reimbursement data) have improved and are increasingly used for epidemiologic studies in France (*7*–*9*). Thus, recent and valid data are available to estimate the population-level health effects of several foodborne pathogens. Such estimates have recently been generated for *Campylobacter* spp. and nontyphoidal *Salmonella* spp. (hereafter referred to as *Salmonella* spp.), the 2 main causes of foodborne bacterial infections in France (*10*). Taking into account this improved knowledge and data availability, we conducted a study to estimate the annual number of illnesses, hospitalizations, and deaths associated with 15 foodborne pathogens in France.

## Methods

Using data sources from 2008–2013, we estimated the number of illnesses, hospitalizations, and deaths in France resulting from 15 foodborne pathogens: 10 bacteria (*Bacillus cereus*, *Campylobacter* spp., *Clostridium botulinum*, *Clostridium perfringens*, Shiga-toxin–producing *Escherichia coli* [STEC], *Listeria monocytogenes*, *Salmonella* spp*., Shigella* spp., *Staphylococcus aureus*, *Yersinia* spp.); 3 viruses (hepatitis A virus, hepatitis E virus, norovirus); and 2 parasites (*Taenia saginata*, *Toxoplasma gondii*). We used France’s 2010 census population (62,765,235 persons) for the estimates. 

We used different statistical models, depending on the most suitable data available for each pathogen, with many inputs to estimate the number of illnesses, hospitalizations, and deaths (Technical Appendix Table 1). For most proportions we defined a lower and upper bound and a beta distribution with 2 parameters derived from a method of moments, assuming a mean *m* = (lower + upper bound)/2 and an SD = (upper bound − *m*)/2 (*11*). We used lognormal probability distributions for model inputs derived from a national survey on acute gastroenteritis in France (*5*) and for the annual numbers of reported illnesses, hospitalizations, and deaths. For final estimates, we multiplied the distributions by using Monte Carlo simulation (10,000 iterations) with R version 3.3.2 (*12*). We report median values and use ranges between the 5th and 95th percentiles of the output distribution to define a 90% credible interval (CrI_90%_).

### Illnesses

To estimate the numbers of illnesses, we obtained surveillance data from the mandatory notification system (*C. botulinum*, *L. monocytogenes*, hepatitis A virus, and foodborne disease outbreaks) and from national reference laboratories and their laboratory surveillance networks (*C. botulinum, Campylobacter* spp., STEC, *L. monocytogenes*, *Salmonella* spp., *Shigella* spp., *Yersinia* spp., hepatitis A virus, hepatitis E virus, and *T. gondii*). Inclusion in these surveillance systems implies that the ill person sought medical care, had laboratory testing prescribed, and had a specimen submitted for laboratory testing and that the laboratory identified the pathogen and reported the positive result to the surveillance system. These steps can be summarized into 2 multiplication factors: an underreporting factor defined as the match between the total number of laboratory-confirmed illnesses and the number of laboratory-confirmed illnesses reported to the surveillance system; and an underdiagnosis factor taking into account the proportion of cases that were not laboratory-confirmed because the patient did not seek medical advice or was misdiagnosed. We took both multiplication factors into account to estimate the number of illnesses from mandatory notification data and national reference laboratory data.

Previously published parameters for estimating the number of *Campylobacter* spp.– and *Salmonella* spp.–associated illnesses (*10*) were used as a proxy to estimate the level of underdiagnosis for *Yersinia* spp. (using *Campylobacter* spp. data) and *Shigella* spp. (using *Salmonella* spp. data). For *C. botulinum* and *L. monocytogenes,* we assumed that 80%–100% of the cases were in persons who sought medical care and had laboratory-confirmed diagnoses. To account for underreporting, we conducted ad hoc laboratory surveys for *Campylobacter* spp., *Salmonella* spp., *Shigella* spp., and *Yersinia* spp., and we conducted a capture–recapture study for *L. monocytogenes*. 

In France, cases of *B. cereus*, *S. aureus,* and *C. perfringens* infection are notified only through mandatory notification of point-source foodborne disease outbreaks. For these pathogens, we assumed that the multiplier between the number of confirmed outbreak cases and the number of community cases of foodborne origin would be similar to that estimated for *Salmonella* spp. We estimated the number of illness caused by *T. gondii* and hepatitis A and E viruses from seroprevalence data and the number of illnesses caused by *T. saginata* from health insurance reimbursement data for niclosamide (a drug used to treat tapeworm infestation). We used data from the literature to estimate the number of illnesses caused by STEC. To estimate the number of norovirus cases, we applied a proportion (14%–22%) of norovirus-associated acute gastroenteritis cases to the annual number of acute gastroenteritis illnesses in France (Table 1). This proportion was based on findings from a 2008–2009 community study in the United Kingdom (*13*) and a meta-analysis of 175 studies published during 1990–2014 (*14*). Model inputs used for each pathogen are presented in Technical Appendix Table 1.

**Table 1:** Data sources used to estimate the number of pathogen-specific illnesses, France, 2008–2013

Pathogen	Data source
*Bacillus cereus*	Surveillance
*Campylobacter* spp.	Surveillance
*Clostridium botulinum*	Surveillance
*Clostridium perfringens*	Surveillance
Hepatitis A virus	Seroprevalence
Hepatitis E virus	Seroprevalence
*Listeria monocytogenes*	Surveillance
Norovirus	Literature and national telephone survey
*Salmonella* spp., nontyphoidal	Surveillance
Shiga toxin–producing *Escherichia coli*	Literature
*Shigella* spp.	Surveillance
*Staphylococcus aureus*	Surveillance
*Taenia saginata*	Health insurance reimbursement data
*Toxoplasma gondii*	Seroprevalence
*Yersinia* spp.	Surveillance

### Hospitalizations

We used the French Hospital Information System (FHIS) as the main data source for estimating the number of hospitalizations. The system is a national database of hospital records that contains sociodemographic information (age, sex, and residence area) and medical information (main cause for admission, concurrent medical conditions, modes of admission, and discharge) (*10*). Diseases are coded according to the International Classification of Diseases, 10th revision (ICD-10; http://www.who.int/classifications/icd/en/). We extracted all hospital records with a patient discharge date during January 2008–December 2013 and containing an ICD-10 code of interest as the main cause for admission or as a concurrent medical condition.

We used the number of hospital records with pathogen-specific ICD-10 codes to estimate the annual number of hospitalizations for 8 pathogens, 4 of which cause acute gastroenteritis (Table 2). We did not redistribute records with only unspecified gastroenteritis codes to the 8 pathogens, but we did correct for undercapture, taking into account the proportion of fecal samples tested for each pathogen and the sensitivity of fecal culture. When data were available, we compared trends over time and patient age and sex distributions of the hospital data with surveillance data from the national reference laboratories (*Campylobacter* spp., *Salmonella* spp., *Shigella* spp., *Yersinia* spp., and hepatitis E virus) and with mandatory notification data (hepatitis A virus).

**Table 2:** Methods used to estimate the number of pathogen-specific hospitalizations, France, 2008–2013*

Pathogen	Method
*Bacillus cereus*	Proportion of hospitalizations for AG applied to annual no. of illnesses for the pathogen
*Campylobacter* spp.	Annual no. persons hospitalized with a specific ICD-10 code in FHIS
*Clostridium botulinum*	Mandatory notification data
*Clostridium perfringens*	Proportion of hospitalizations for AG applied to annual no. of illnesses for the pathogen
Hepatitis A virus	Annual no. persons hospitalized with a specific ICD-10 code in FHIS
Hepatitis E virus	Annual no. persons hospitalized with a specific ICD-10 code in FHIS
*Listeria monocytogenes*	Mandatory notification data
Norovirus	Proportion of hospitalizations for AG applied to annual no. of illnesses for the pathogen
*Salmonella* spp., nontyphoidal	Annual no. persons hospitalized with a specific ICD-10 code in FHIS
Shiga toxin–producing *Escherichia coli*	*Salmonella* spp. and *Campylobacter* spp. data used as a proxy
*Shigella* spp.	Annual no. persons hospitalized with a specific ICD-10 code in FHIS
*Staphylococcus aureus*	Proportion of hospitalizations for AG applied to annual no. of illnesses for the pathogen
*Taenia saginata*	Annual no. persons hospitalized with a specific ICD-10 code in FHIS
*Toxoplasma gondii*	Annual no. persons hospitalized with a specific ICD-10 code in FHIS
*Yersinia* spp.	Annual no. persons hospitalized with a specific ICD-10 code in FHIS

*AG, acute gastroenteritis; FHIS, French Hospital Information System; ICD-10, International Classification of Diseases, 10th Revision (http://www.who.int/classifications/icd/en/).

We used the number of hospital records with acute gastroenteritis–associated ICD-10 codes (A00–A06.2 and A06.9–A09.9) to estimate the annual number of persons hospitalized for acute gastroenteritis. We then divided that number by the total number of persons with acute gastroenteritis to estimate the percentage of those persons who were hospitalized (0.58%–0.75%) (Technical Appendix Table 1). For norovirus, *B. cereus*, *C. perfringens*, and *S. aureus,* we applied the proportion of hospitalizations for acute gastroenteritis to the annual number of illnesses for each pathogen to estimate the annual number of hospitalizations. For STEC, we used the proportion of hospitalizations estimated for *Salmonella* spp. and *Campylobacter* spp. as a proxy. For *C. botulinum* and *L. monocytogenes*, we used surveillance data from the mandatory notification system (Table 2).

### Deaths

We explored death certificate data from the French national mortality database (**Institut National de la Santé et de la Recherche Médicale**, CépiDc [Epidemiology Center on Medical Causes of Death]) and data from FHIS to estimate the number of foodborne illness–associated deaths. For both data sources, we extracted all records for 2008–2013 with an ICD-10 code of interest as the main cause of death or hospitalization or as a concurrent medical condition. Compared with data from FHIS, death certificates contained fewer pathogen-specific ICD-10 codes; therefore, we used the hospital information system data as the main data source for estimating the number of deaths.

To estimate the number of deaths from *Campylobacter* spp., *Salmonella* spp., *Shigella* spp., *Yersinia* spp., hepatitis A and E viruses, *T. saginata*, and *T. gondii* infections, we used the number of hospital records with a pathogen-specific ICD-10 code and death shown as the mode of discharge*.* To estimate the number of norovirus-associated deaths, we applied the proportion of deaths among hospitalized case-patients with an ICD-10 code associated with viral gastroenteritis (ICD-10 codes A08.0–A08.4) to the annual number of hospitalizations for norovirus (0.18%–0.30%; Technical Appendix Table 1). This proportion was also used as a proxy to estimate the number of deaths from *B. cereus*–, *C. perfringens*–, and *S. aureus*–associated hospitalizations. For *C. botulinum* and *L. monocytogenes*, we used mandatory notification data to estimate the number of deaths (Table 3).

**Table 3:** Methods used to estimate the number of pathogen-specific deaths, France, 2008–2013*

Pathogen	Method
*Bacillus cereus*	Hospital discharge data with viral gastroenteritis–associated ICD-10 codes
*Campylobacter* spp.	Hospital discharge data with pathogen-specific ICD-10 codes
*Clostridium botulinum*	Mandatory notification data
*Clostridium perfringens*	Hospital discharge data with viral gastroenteritis–associated ICD-10 codes
Hepatitis A virus	Hospital discharge data with pathogen-specific ICD-10 codes
Hepatitis E virus	Hospital discharge data with pathogen-specific ICD-10 codes
*Listeria monocytogenes*	Mandatory notification data
Norovirus	Hospital discharge data with viral gastroenteritis–associated ICD-10 codes
*Salmonella* spp., nontyphoidal	Hospital discharge data with pathogen-specific ICD-10 codes
Shiga toxin–producing *Escherichia coli*	*Salmonella* spp. and *Campylobacter* spp. data used as a proxy
*Shigella* spp.	Hospital discharge data with pathogen-specific ICD-10 codes
*Staphylococcus aureus*	Hospital discharge data with viral gastroenteritis–associated ICD-10 codes
*Taenia saginata*	Hospital discharge data with pathogen-specific ICD-10 codes
*Toxoplasma gondii*	Hospital discharge data with pathogen-specific ICD-10 codes
*Yersinia* spp.	Hospital discharge data with pathogen-specific ICD-10 codes

*ICD-10, International Classification of Diseases, 10th Revision (http://www.who.int/classifications/icd/en/).

### Foodborne Transmission

To estimate the number of foodborne illnesses and associated hospitalizations and deaths, we applied a pathogen-specific proportion of foodborne transmission (Technical Appendix Table 2). For 11 of the 15 pathogens, we used estimates published in the United States in 2011 (*15*). For norovirus and hepatitis A virus, data from more recent studies were used (*16*,*17*). For hepatitis E virus and *T. saginata*, the proportions of foodborne transmission were estimated on the basis of discussions with experts from the French Public Health Agency.

## Results

Overall, the pathogens included in our study accounted for 4.9 million cases of illness (CrI_90%_ 4.2–6.2 million), 42,500 hospitalizations (CrI_90%_ 37,242–50,526), and 368 deaths (CrI_90%_ 335–486) each year in France. Of those 4.9 million cases, 1.5 million were caused by foodborne pathogens (CrI_90%_ 1.28–2.23 million), of which 880,500 (59%) were caused by bacteria; 579,500 (38%) by viruses; and 45,000 (3%) by parasites. These foodborne illnesses led to 17,281 hospitalizations (CrI_90%_ 15,520–20,785) and 248 deaths (CrI_90%_ 223–350).

Norovirus ranked first as the cause of foodborne illnesses (34%), third as a cause for foodborne illness–associated hospitalizations (20%), and seventh as a cause of foodborne illness–associated deaths (3%). *Salmonella* spp. ranked third as the cause of foodborne illnesses (12%), second as a cause for hospitalization (24%), and first as a cause of death (27%). *L monocytogenes* ranked second (26%), before *Campylobacter* spp. (17%), as a cause of foodborne illness–associated deaths (Technical Appendix Table 2).

## Discussion

We estimated the population-level number of illnesses, hospitalizations, and deaths in France caused by 15 pathogens with the potential for foodborne transmission. *Campylobacter* spp., *Salmonella* spp., and norovirus were responsible for 73% of all foodborne illnesses and 76% of all associated hospitalizations. The pathogens that cause most foodborne illnesses or hospitalizations are not necessarily those that cause the most deaths: *L. monocytogenes* caused <0.1% of all foodborne illnesses but ranked second as a cause of foodborne illness–associated deaths, just behind S*almonella* spp. 

We used different approaches, depending on the most suitable data that were available, to generate estimates. We could not easily compare our results with previous estimates from France (*2*) and other countries because of different data sources, assumptions, and methods. Nevertheless, recent estimates of the burden of foodborne illnesses in the European region also indicated that the 3 most frequent causes of foodborne illness were norovirus (ranked first), *Campylobacter* spp. (second), and *Salmonella* spp. (third) (*1*). These pathogens were also among the leading causes of foodborne illnesses and hospitalizations in North America (*15*,*18*) and Oceania (*19*,*20*). *Salmonella* spp. and *L*. *monocytogenes* accounted for ≈50% of all foodborne illness–associated deaths in France, and were also responsible for most foodborne illness–associated deaths in other high-income countries (*1*,*15*,*18*–*20*).

We estimated the number of most pathogen-specific illnesses by using laboratory-based surveillance data corrected for underreporting and underdiagnosis, and we used well-documented estimates for *Campylobacter* spp. and *Salmonella* spp. (*10*). We assumed that the parameters regarding healthcare-seeking behavior and laboratory practice for *Yersinia* spp. and *Shigella* spp. were similar to those for *Campylobacter* spp. and *Salmonella* spp., respectively. The validity of these assumptions is difficult to explore; further studies would be needed to produce more robust estimates of the true level of underdiagnosis for these 2 pathogens in France.

For *B. cereus*, *C. perfringens*, and *S. aureus*, we assumed that the multiplier between the number of outbreak cases and the number of foodborne illnesses would be similar to that for *Salmonella* spp. An alternative approach for *C. perfringens* would have been to apply a proportion of acute gastroenteritis cases by this pathogen estimated in the United Kingdom (0.3–1.7%) (*13*) to the annual number of acute gastroenteritis illnesses in France. This approach would result in an estimate (CrI_90%_ 84,450–278,964) within the range of the estimate in our study. The estimates for *B. cereus*, *C. perfringens*, and *S. aureus* indicate that the effect of these pathogens in terms of foodborne illnesses appears to be high in France. However, only foodborne illness outbreak data were available to estimate the number of illnesses for these pathogens, and more data are needed to confirm our estimates.

We included hepatitis E virus in our study because, in France, indigenous cases of hepatitis E have been shown to be associated with foodborne transmission, particularly through consumption of products containing undercooked or raw pork liver (*21*,*22*). We estimated the number of hepatitis E cases in France from a seroprevalence study conducted in 2013, and the proportion of cases caused by foodborne transmission was assumed to be between 75% and 100%. Further studies, in particular on the proportion of foodborne transmission of hepatitis E in France, are needed to confirm these estimates. 

Our use of seroprevalence and health insurance drug reimbursement data to estimate the numbers of *T. gondii*– and *T. saginata*–associated foodborne illnesses was similar to methods previously used in France (*2*). Our results indicated a decrease in the number of foodborne illnesses over the past decade (from 51,600 to 12,000 cases for *T. gondii* and from 64,500 to 33,000 cases for *T. saginata*). These decreases may be explained by fewer exposures to the parasites (*23*), by changes in food habits, and by improved hygiene practices in meat production. For *T. saginata*, the number of illnesses may be underestimated because the decrease might also be explained by a shift of treatment from niclosamide to praziquantel for this infection over the past decade in France.

We estimated the number of illnesses caused by norovirus by applying a proportion of acute gastroenteritis cases caused by this pathogen to the annual number of acute gastroenteritis illnesses in France. The final estimate for France is lower than that for other countries that used a similar method (*15*,*18*), primarily because of a lower estimated incidence of acute gastroenteritis in France (*5*) but also because we used a lower proportion of foodborne norovirus transmission (12%–16%) on the basis of an extensive study published in 2015 (*16*). Despite these differences and their effect on the final estimate, norovirus ranked first in terms of foodborne illnesses in France and appears to be a key foodborne cause of acute gastroenteritis.

The FHIS was our main data source for estimating numbers of hospitalizations and deaths associated with the 15 pathogens in our study. The relevance of this data source may be questioned because of limitations in diagnosis accuracy and in consistency of disease coding. For most of the pathogens, we estimated the number of hospitalizations by using the number of hospital records with specific ICD-10 codes. We compared trends over time and age and sex distributions of the hospital data with surveillance data from the national reference laboratories and with mandatory notification data. Trends and distributions were similar between the different data sources, supporting the use of FHIS data to estimate the number of hospitalizations. For *Campylobacter* spp., *Salmonella* spp., *Yersinia* spp., and *Shigella* spp., we corrected the number of hospitalizations and deaths for underdiagnosis, taking into account a proportion of fecal samples tested for each pathogen and the sensitivity of fecal culture. However, for the other pathogens, no specific underdiagnosis multiplier could be estimated and, therefore, the estimates presented in this study are probably conservative. An overestimation is also possible if the pathogen of interest did not cause the illness that led to the hospitalization but was, nevertheless, coded as a concurrent medical condition.

A high number of hospitalizations due to acute gastroenteritis were reported in the FHIS without a specific ICD-10 code because not all hospitalized patients were systematically tested for all pathogens that cause acute gastroenteritis. We used the proportion of hospitalizations for acute gastroenteritis as a proxy to estimate the number of hospitalizations for norovirus, *B. cereus*, *C. perfringens*, and *S. aureus* because testing for these pathogens is infrequently performed in France and because these pathogens cause illnesses with similar symptoms and severity. This proportion (0.58%–0.75%) is lower than that estimated for *Campylobacter* spp. (0.9%–1.9%) and for *Salmonella* spp. (1.2%–3.6%), which is plausible considering that illness caused by *B. cereus*, *C. perfringens*, and *S. aureus* is less severe than that caused by *Campylobacter* spp. and *Salmonella* spp. Data sources described in the literature to estimate the number of hospitalizations for norovirus, *B. cereus*, *C. perfringens*, and *S. aureus* infections include hospital discharge data and data from foodborne disease outbreaks (*15*,*18*,*19*,*24*,*25*). Estimating the number of hospitalizations for these pathogens is challenging, and these different methodologic approaches have a major effect on the final estimate. For norovirus, despite differences in methodology and healthcare systems, our estimate (all modes of transmission) of the number of hospitalizations was in the same range as those estimated in North America (*24*,*25*) and in the Netherlands (*26*).

Data to estimate the number of deaths associated with foodborne illnesses are scarce and difficult to obtain. We explored death certificate data but decided not to use that source because few records contained pathogen-specific ICD-10 codes. Hospital discharge data were the only or the most reliable data source available to estimate the number of deaths for most pathogens included in this study. However, deaths may occur after hospitalization discharge or without hospitalization at all. Therefore, our estimates are uncertain and are probably underestimated, even though we did not take into account the possibility that underlying concurrent conditions, not foodborne pathogens, may have caused or contributed to death.

As pointed out in the literature, difficulties in accurately determining the proportion of foodborne pathogen transmission is a key factor contributing to the uncertainty of foodborne illness estimates (*15*,*27*). Different methodologic approaches, such as epidemiologic and microbiologic approaches, intervention studies, and expert elicitation, have been used to estimate the proportion of foodborne transmission (*15*,*28*–*32*). Overall, in high-income countries, foodborne transmission has been considered a major transmission route for several bacterial pathogens (*B. cereus*, *Campylobacter* spp., *C. perfringens*, *L. monocytogenes*, *Salmonella* spp., *S. aureus*) and a minor transmission route for norovirus and hepatitis A virus. Nevertheless, comparison of the estimates by using expert elicitation shows greater variability and higher uncertainties, depending on how the experts were recruited, the expert panel size, or the elicitation method used (*27*,*33*). We decided to use the proportion of foodborne transmission published in the United States in 2011 (*15*) as these proportions were based on epidemiologic and microbiologic data rather than expert elicitation. It is possible that food consumption patterns and frequency and type of microbiologic contamination differ between the United States and France and may influence pathogen exposure, resulting in a different proportion of foodborne pathogen transmission in the 2 countries. Further research is needed to obtain specific source attribution estimates for France.

The 15 foodborne pathogens in our study were selected on the basis of their perceived public health significance, their occurrence in France, and the availability of a minimum of data. Other known pathogens with potential foodborne transmission exist (e.g., other non-STEC pathogenic *E.coli*, rotavirus, and *Cryptosporidium* spp.), and the total numbers of foodborne illnesses and associated hospitalizations and deaths presented in this study are likely conservative.

We took into account new data sources that allowed for accurate estimates of foodborne illnesses and associated hospitalizations and deaths at the community level in France. Our estimates entail several assumptions, and a high degree of uncertainty remains for some of them. Our estimates indicate that substantial numbers of foodborne pathogen–associated illnesses, hospitalizations, and deaths occur each year in France, necessitating the prioritization of prevention and control strategies by food safety policymakers. We did not specifically consider the effect of sequelae linked to these illnesses when generating our estimates. Thus, our findings capture only part of the overall effect of foodborne infections, and they clear the way for further research on the public health burden of foodborne pathogens in France, taking into account complications and sequelae.

Technical Appendix Additional information on estimated annual numbers of foodborne pathogen–associated illnesses, hospitalizations, and deaths, France, 2008–2013.
