# Supplementary material for: Estimated Annual Numbers of Foodborne Pathogen–Associated Illnesses, Hospitalizations, and Deaths, France, 2008–2013
Source: Emerg Infect Dis. 2017 Sep;23(9):1486–92. doi: 10.3201/eid2309.170081 (PMC5572882; doi:10.3201/eid2309.170081)
Supplement: Technical Appendix — Additional information on estimated annual numbers of foodborne pathogen–associated illnesses, hospitalizations, and deaths, France, 2008–2013. [file 17-0081-Techapp-s1.pdf]

Article DOI: https://doi.org/10.3201/eid2309.170081

Estimated Annual Numbers of Foodborne Disease–Associated Illnesses, Hospitalizations, and Deaths, France, 2008–2013

Technical Appendix

Technical Appendix Table 1. Model inputs to estimate the number of illnesses, hospitalizations and deaths for 15 foodborne pathogens, France, 2008–2013

Model input	Data source(s)	Distribution	Data for model input
Acute gastroenteritis (AG)			
Acute gastrointestinal illnesses	Estimated rate of acute gastrointestinal illness per person and per year based on a population-based study in 2009/2010 in France (*1*)	log normal	meanlog: -1.109, sdlog: 0.0696
Population at risk	2010 French census population estimate	Fixed	62,765,235
Acute gastrointestinal illnesses	Number estimated using the two parameters above	Outcome	5% value: 18,454,814, median value: 20,725,683, 95% value: 23,213,292
Acute gastrointestinal hospitalizations	Annual number of patients with AG related ICD-10 codes (A00 – A06.2, A06.9 – A09.9) reported in the French hospital information system (2008-2013)	log normal	meanlog: 11.824, sdlog: 0.039
Proportion hospitalized	Proportion estimated using the two parameters above	Outcome	5% value: 0.00577, median value: 0.00659, 95% value: 0.00752
Bacillus cereus			
Reported illnesses	Annual number of reported foodborne outbreak cases reported to the French national public health agency between 2008 and 2013	log normal	meanlog: 5.690, sdlog: 0.179
Underreporting	*Salmonella* spp. data used as a proxy (2008-2013) : annual number of reported foodborne outbreak cases (min 509 - max 1 066) divided by the annual number of food-related laboratory confirmed cases min 13 872 – max 15 074)	Beta	α: 25.11, β: 427.49
Test sensitivity	Sensitivity of stool culture for *Salmonella* spp. used as a proxy	Beta	α: 71.25, β: 3.75
Laboratory testing	The proportion of stool samples tested for *Salmonella* spp. used as a proxy	Beta	α: 71.25, β: 3.75
Specimen submission	Proportion of stool samples prescribed for *Salmonella* spp. used as a proxy	Beta	α: 21.36, β: 108.08
Medical care seeking if long duration of illness	Proportion of cases of AG with a duration of illness longer than 5 days that consulted for their illness, estimated from a national telephone survey conducted in 2009-2010 (*1*)	log normal	meanlog: -0.346, sdlog: 0.141
Proportion of cases with a long duration of illness	Proportion of *Salmonella* spp. cases with a duration of illness longer than 5 days used as a proxy	Beta	α: 195.43, β: 203.41
Medical care seeking if medium duration of illness	Proportion of cases of AG with a duration of illness of 3-5 days that consulted for their illness, estimated from a national telephone survey conducted in 2009-2010 (*1*)	log normal	meanlog: -0.865, sdlog: 0.142
Proportion of cases with a medium duration of illness	Proportion of *Salmonella* spp. cases with a duration of illness of 3-5 days used as a proxy	Beta	α: 186.85, 210.71
Medical care seeking if short duration of illness	Proportion of cases of AG with a duration of illness of less than 3 days that consulted for their illness, estimated from a national telephone survey conducted in 2009-2010 (*1*)	log normal	meanlog: -1.708, sdlog: 0.216
Proportion of cases with a short duration of illness	Proportion of *Salmonella* spp. cases with a duration of illness of less than 3 days used as a proxy	Beta	α: 383.96, β: 9,215.04
Proportion hospitalized	Proportion estimated for acute gastroenteritis used as a proxy (*cf.* table acute gastroenteritis in this appendix)	Beta	α: 229.15.11, β: 34,255.89
Proportion of hospitalized patients who died	Proportion estimated for norovirus used as a proxy (*cf.* table norovirus in this appendix)	Beta	α: 63.84, β: 26,537.82
Proportion foodborne	Only foodborne outbreak cases reported in France. Proportion estimated to be 100%, similar to US estimates published in 2011 (*2*)	Fixed	100%
Campylobacter spp.			
Reported illnesses	Annual number of cases of *Campylobacter* spp. reported by the national reference center between 2008 and 2013	log normal	meanlog: 8.436, sdlog: 0.157
Underreporting	Completeness of case reporting to the national reference center, estimated from a national laboratory survey carried out in 2010 (unpublished data, French national public health agency (*3*))	Beta	α: 348.34, β: 1176.13
Test sensitivity	Sensitivity of stool culture for *Campylobacter* spp., estimated by the national reference center in 2009 (*4*)	Beta	α 229.8, β: 153.2

Model input	Data source(s)	Distribution	Data for model input
Laboratory testing	Proportion of stool samples tested for *Campylobacter* spp., estimated from a national laboratory survey carried out in 2010 (unpublished data, French national public health agency (*3*))	Beta	α: 81.121, β: 44.648
Specimen submission	Proportion of stool samples prescribed for cases of *Campylobacter* spp. that consulted a general practitioner (GP), estimated from a survey among GPs carried out in 2013-2014 (*5*)	Beta	α: 17.792, β: 83.875
Medical care seeking if long duration of illness	Proportion of cases of AG with a duration of illness longer than 5 days that consulted for their illness, estimated from a national telephone survey conducted in 2009-2010 (1)	log normal	meanlog: -0.346, sdlog: 0.141
Proportion of cases with a long duration of illness	Proportion of *Campylobacter* spp. cases with a duration of illness longer than 5 days, estimated from a national case-control study (2002-2004) (*6*)	Beta	α: 118.63, β: 48.45
Medical care seeking if medium duration of illness	Proportion of cases of AG with a duration of illness of 3-5 days that consulted for their illness, estimated from a national telephone survey conducted in 2009-2010 (*1*)	log normal	meanlog: -0.865, sdlog: 0.142
Proportion of cases with a medium duration of illness	Proportion of *Campylobacter* spp. cases with a duration of illness of 3-5 days, estimated from a national case-control (2002-2004) (*6*)	Beta	Α: 236.25, β: 638.75
Medical care seeking if short duration of illness	Proportion of cases of AG with a duration of illness less than 3 days that consulted for their illness, estimated from a national telephone survey conducted in 2009-2010 (*1*)	log normal	meanlog: -1.708, sdlog: 0.216
Proportion of cases with a short duration of illness	Proportion of *Campylobacter* spp. cases with a duration of illness less than 3 days, estimated from a national case-control study (2002-2004) (*6*)	Beta	α: 391.98, β: 19207.02
Reported hospitalizations	Annual number of patients hospitalized with an ICD-10 code of campylobacteriosis (A04.5) reported in the French hospital information system (2008-2013)	log normal	meanlog: 8.032, sdlog: 0.168
Reported deaths	Annual number of patients hospitalized with an ICD-10 code of campylobacteriosis (A04.5) reported in the French hospital information system (2008-2013) with "death" coded as the mode of discharge	log normal	meanlog: 3.136, sdlog: 0.261
Laboratory testing: hospitalizations/deaths	Proportion of stool samples tested for *Campylobacter* spp. in hospital laboratories estimated from a national laboratory survey carried out in 2010 (unpublished data, French national public health agency)	Beta	α: 46.326, β: 16.277
Test sensitivity: hospitalizations/deaths	Sensitivity of stool culture for *Campylobacter* spp., estimated by the National Reference Center in 2009 (*4*)	Beta	α: 229.8, β: 153.2
Proportion foodborne	Estimated to be between 73 and 86% based on US estimates published in 2011 (*2*)	Beta	α: 121.78, β: 31.426
Clostridium botulinum			
Reported illnesses	Annual number of cases reported by the national reference center – mandatory notification surveillance in France between 2008 and 2013	log normal	meanlog: 2.890, sdlog: 0.3889
Underreporting	Completeness of case reporting to the national reference center – mandatory notification surveillance was assumed to be between 90 and 100%	Beta	α: 71.25, β: 3.75
Test sensitivity, laboratory testing, specimen submission, medical care seeking	Cases are reported through mandatory notification that is based on their clinical symptoms. They are reported even in absence of laboratory confirmation. Proportion assumed to be between 80 and 100%.	Beta	α: 31.5, β: 3.5
Proportion hospitalized	Proportion of hospitalization among the cases reported by mandatory notification surveillance between 2008 and 2013 (85 – 100%)	Beta	α: 44.708, β: 3.625
Deaths	Mean annual number of deaths reported by mandatory notification surveillance between 2008 and 2013	log normal	meanlog: -1.204, sdlog: 1.567
Proportion foodborne	All cases reported by mandatory notification surveillance are assumed food-related (no other mode of transmission documented). Proportion similar to US estimates published in 2011 (*2*)	Fixed	100%
Clostridium perfringens			
Reported illnesses	Annual number of reported foodborne outbreak cases reported to the French national public health agency between 2008 and 2013	log normal	meanlog: 6.240, sdlog: 0.370
Underreporting	*Salmonella* spp. data used as a proxy (2008-2013) : annual number of reported foodborne outbreak cases (min 509 - max 1 066) divided by the annual number of food-related laboratory confirmed cases min 13 872 – max 15 074)	Beta	α: 25.11, β: 427.49
Test sensitivity	Sensitivity of stool culture for *Salmonella* spp. used as a proxy	Beta	α: 71.25, β: 3.75
Laboratory testing	The proportion of stool samples tested for *Salmonella* spp. used as a proxy	Beta	α: 71.25, β: 3.75
Specimen submission	Proportion of stool samples prescribed for *Salmonella* spp. used as a proxy	Beta	α: 21.36, β: 108.08
Medical care seeking if long duration of illness	Proportion of cases of AG with a duration of illness longer than 5 days that consulted for their illness, estimated from a national telephone survey conducted in 2009-2010 (*1*)	log normal	meanlog: -0.346, sdlog: 0.141
Proportion of cases with a long duration of illness	Proportion of *Salmonella* spp. cases with a duration of illness longer than 5 days used as a proxy	Beta	α: 195.43, β: 203.41
Medical care seeking if medium duration of illness	Proportion of cases of AG with a duration of illness of 3-5 days that consulted for their illness, estimated from a national telephone survey conducted in 2009-2010 (*1*)	log normal	meanlog: -0.865, sdlog: 0.142
Proportion of cases with a medium duration of illness	Proportion of *Salmonella* spp. cases with a duration of illness of 3-5 days used as a proxy	Beta	α: 186.85, 210.71

Model input	Data source(s)	Distribution	Data for model input
Medical care seeking if short duration of illness	Proportion of cases of AG with a duration of illness of less than 3 days that consulted for their illness, estimated from a national telephone survey conducted in 2009-2010 (*1*)	log normal	meanlog: -1.708, sdlog: 0.216
Proportion of cases with a short duration of illness	Proportion of *Salmonella* spp. cases with a duration of illness of less than 3 days used as a proxy	Beta	α: 383.96, β: 9,215.04
Proportion hospitalized	Proportion estimated for acute gastroenteritis used as a proxy (*cf*. table acute gastroenteritis in this appendix)	Beta	α: 229.15.11, β: 34,255.89
Proportion of hospitalized patients who died	Proportion estimated for norovirus used as a proxy (*cf*. table norovirus in this appendix)	Beta	α: 63.84, β: 26,537.82
Proportion foodborne	Only foodborne outbreak cases reported in France. Proportion estimated to be 100%, similar to US estimates published in 2011 (*2*)	Fixed	100%
Escherichia coli, Shiga-toxin-producing (STEC)			
Incidence	Estimated annual rate per 100,000 from published studies in Germany (*7*), the Netherlands (*8*) and the European region (*9*)	log normal	meanlog: 3.664, sdlog: 0.462
Population at risk	2010 French census population estimate	Fixed	62,765,235
Hospitalizations	Proportion of hospitalization estimated for *Salmonella* spp. and *Campylobacter* spp. illnesses used as a proxy	Beta	α: 9.716, β: 434.958
Proportion of hospitalized patients who died	Proportion of hospitalized patients who died estimated for *Salmonella* spp. and *Campylobacter* spp. used as a proxy	Beta	α: 19.663, β: 1,675.408
Proportion foodborne	Estimated to be between 59 and 87% based on US estimates published in 2011 (*2*)	Beta	α: 28.634, β: 10.591
Hepatitis A virus (HAV)			
Reported illnesses	Annual number of domestic symptomatic cases of hepatitis A virus (HAV) reported to the French national public health agency between 2008 and 2013 (mandatory notifiable disease)	log normal	meanlog: 7.034, sdlog: 0.158
Sensibility	Sensibility the French surveillance system (6.55-7.3%), estimated from age specific incidence and seroprevalence data using a catalytic model that has been used to estimate HAV incidence in the USA (*10*) and in Canada (*11*)*	Beta	α: 1,283.548, β: 17,248.77
Hospitalizations	Annual number of patients hospitalized with an ICD-10 code related to HAV (B15.0 or B15.9) reported in the French hospital information system (2008-2013)	log normal	meanlog: 7.354, sdlog: 0.196
Deaths	Annual number of patients hospitalized with an ICD-10 code related to HAV (B15.0 or B15.9) reported in the French hospital information system (2008-2013) with "death" coded as mode of discharge	log normal	meanlog: 3.401, sdlog: 0.133
Proportion foodborne	Proportion based on a study conducted in the Netherlands (2008-2010 data) (*12*)	Fixed	16%
Hepatitis E virus (HEV)			
Number of hepatitis E virus (HEV) positive blood donors (A)	Number of blood donors RNA positive (PCR) estimated from a seroprevalence study conducted in 2013 (*13*)	log normal	meanlog: 6.670, sdlog: 0.197
Annual number of blood donors (B)	Annual number of blood donors in France (*14*)	Fixed	2,941,624
Viremia (V)	Duration of viremia of HEV assumed to be between 21 and 35 days	log normal	meanlog: 3.332, sdlog: 0.1275
Incidence	Annual incidence rate of hepatitis E virus (cases per 1,000 inhabitants) estimated using the following formula: (A)/[(B)*(V/365)]	outcome	5% value: 2.38, median value: 3.50, 95% value: 5.17
Population at risk	2010 French census population estimate	Fixed	62,765,235
Symptomatic illnesses	Proportion of symptomatic infections (29-33%) estimated from two outbreaks investigations conducted on a cruise ship in 2008 (*15*) and on a French island in 2013 (*16*)	Beta	α: 662.78, β: 1,475.22
Hospitalizations	Annual number of patients hospitalized with an ICD-10 code related to HEV (B17.2) reported in the French hospital information system (2013-2014)	log normal	meanlog: 6.303, sdlog: 0.0073
Deaths	Mean annual number of patients hospitalized with an ICD-10 code related to HEV (B17.2) reported in the French hospital information system (2013-2014) with "death" coded as mode of discharge	log normal	meanlog: 2.9957, sdlog: 0.05
Proportion foodborne	Proportion assumed to be between 75 and 100%	Beta	α: 23.625, β: 3.375
Listeria monocytogenes			
Reported illnesses	Annual number of cases reported by the national reference center – mandatory notification surveillance in France between 2008 and 2013	log normal	meanlog: 5.740, sdlog: 0.113
Underreporting	Completeness of case reporting for the 2008-2014 period estimated from a capture-recapture study using national reference center data, mandatory notification data and data from a laboratory network of six severe invasive bacterial diseases including listeriosis (unpublished data, French Public Health Agency)	Beta	α: 4,140.9, β: 674.1
Test sensitivity, laboratory testing, specimen submission, medical care seeking	Proportion assumed to be between 80 and 100%.	Beta	α: 31.5, β: 3.5
Hospitalizations	Mean annual number of hospitalizations reported by mandatory notification surveillance between 2008 and 2013	log normal	meanlog: 5.740, sdlog: 0.113
Deaths	Mean annual number of deaths reported by mandatory notification surveillance between 2008 and 2013	log normal	meanlog: 4.174, sdlog: 0.267
Proportion foodborne	All cases reported by mandatory notification surveillance are assumed food-related (no other mode of transmission documented). Proportion similar to US estimates published in 2011 (*2*)	Fixed	100%
Norovirus			

Model input	Data source(s)	Distribution	Data for model input
Acute gastrointestinal illnesses	Estimated rate of acute gastrointestinal illness per person and per year based on a population-based study in 2009/2010 in France	log normal	meanlog: -1.109, sdlog: 0.0696
Population at risk	2010 French census population estimate	Fixed	62,765,235
Norovirus illnesses	Proportion of AG due to norovirus (14-22%), estimated from a prospective population-based cohort study in the United Kingdom (*17*,*18*) and a systematic review and meta-analysis (developed countries) (*19*)	Beta	α: 66.24, β: 301.76
Proportion hospitalized	Proportion estimated for acute gastroenteritis used as a proxy (*cf*. table acute gastroenteritis in this appendix)	Beta	α: 229.15.11, β: 34,255.89
Proportion of hospitalized patients who died	Proportion of cases hospitalized with an ICD-10 code related to viral gastroenteritis (ICD-10 codes A08.0 – A08.5) in the French hospital information system (2008-2013) with “death” coded as mode of discharge	Beta	α: 63.884, β: 26,537.82
Proportion foodborne	Proportion (12-16%) based on a study published in 2015 (*20*) that used data from outbreak surveillance systems and from a systematic review of the literature to estimate the proportion of norovirus due to food	Beta	α: 168.42, β: 1,034.58
Salmonella spp.			
Reported illnesses	Annual number of cases of *Salmonella* spp. reported by the national reference center between 2008 and 2013	log normal	meanlog: 9.193, sdlog: 0.052
Underreporting	Completeness of case reporting to the national reference center, estimated from a national laboratory survey carried out in 2009 (*21*)	Beta	α: 479.94, β: 275.87
Test sensitivity	Sensitivity of stool culture for *Salmonella* spp. assumed to be between 90 and 100%	Beta	α 71.25, β: 3.75
Laboratory testing	*Salmonella* spp. is routinely tested on stool samples in France. The proportion of stool samples tested for *Salmonella* spp. is assumed to be between 90 and 100%	Beta	α 71.25, β: 3.75
Specimen submission	Proportion of stool samples prescribed for cases of *Salmonella* spp. that consulted a general practitioner (GP), estimated from a survey among GPs carried out in 2013 – 2014 (*5*)	Beta	α: 21.36, β: 108.08
Medical care seeking if long duration of illness	Proportion of cases of AG with a duration of illness longer than 5 days that consulted for their illness, estimated from a national telephone survey conducted in 2009-2010 (*1*)	log normal	meanlog: -0.346, sdlog: 0.141
Proportion of cases with a long duration of illness	Proportion of *Salmonella* spp. cases with a duration of illness longer than 5 days, estimated from outbreaks investigated during the 2008-2013 period (unpublished data, French national public health agency)	Beta	α: 195.43, β: 203.41
Medical care seeking if medium duration of illness	Proportion of cases of AG with a duration of illness of 3-5 days that consulted for their illness, estimated from a national telephone survey conducted in 2009-2010 (*1*)	log normal	meanlog: -0.865, sdlog: 0.142
Proportion of cases with a medium duration of illness	Proportion of *Salmonella* spp. cases with a duration of illness of 3-5 days, estimated from outbreaks investigated during the 2008-2013 period (unpublished data, French national public health agency)	Beta	α: 186.85, β:210.71
Medical care seeking if short duration of illness	Proportion of cases of AG with a duration of illness less than 3 days that consulted for their illness, estimated from a national telephone survey conducted in 2009-2010 (*1*)	log normal	meanlog: -1.708, sdlog: 0.216
Proportion of cases with a short duration of illness	Proportion of *Salmonella* spp. cases with a duration of illness of less than 3 days, estimated from outbreaks investigated during the 2008-2013 period (unpublished data, French national public health agency	Beta	α: 383.96, β: 9,215.04
Reported hospitalizations	Annual number of patients hospitalized with an ICD-10 code of salmonellosis (A02 – A02.9) reported in the French hospital information system (2008-2013)	log normal	meanlog: 8.341, sdlog: 0.0698
Reported deaths	Annual number of patients hospitalized with an ICD-10 code of salmonellosis (A02 – A02.9) reported in the French hospital information system (2008-2013) with “death” coded as the mode of discharge	log normal	meanlog: 4.2195, sdlog: 0.088
Laboratory testing: hospitalizations/deaths	Proportion of stool samples tested for *Salmonella* spp. in hospital laboratories assumed to be 100%	Fixed	100%
Test sensitivity: hospitalizations/deaths	Sensitivity of stool culture for *Salmonella* spp., assumed to be between 90 and 100%	Beta	α 71.25, β: 3.75
Proportion foodborne	Estimated to be between 91 and 95% based on US estimates published in 2011 (*2*)	Beta	α: 604.5, β: 45.5
Shigella spp.			
Reported illnesses	Annual number of cases of *Shigella* spp. reported by the national reference center between 2008 and 2013	log normal	meanlog: 6.498, sdlog: 0.009
Underreporting	Completeness of case reporting to the national reference center, estimated from a national laboratory survey carried out in 2009	Beta	α: 452.87, β: 348.67
Test sensitivity	Sensitivity of stool culture for *Shigella* spp. assumed to be between 90 and 100%	Beta	α 71.25, β: 3.75
Laboratory testing	*Shigella* spp. is routinely tested on stool samples in France. The proportion of stool samples tested for *Shigella* spp. is assumed to be between 90 and 100%	Beta	α 71.25, β: 3.75
Specimen submission	Proportion of stool samples prescribed *Salmonella* spp. used as a proxy	Beta	α: 21.36, β: 108.08
Medical care seeking if long duration of illness	Proportion of cases of AG with a duration of illness longer than 5 days that consulted for their illness, estimated from a national telephone survey conducted in 2009-2010 (*1*)	log normal	meanlog: -0.346, sdlog: 0.141
Proportion of cases with a long duration of illness	Proportion of *Salmonella* spp.; cases with a duration of illness longer than 5 days used as a proxy	Beta	α: 195.43, β: 203.41

Model input	Data source(s)	Distribution	Data for model input
Medical care seeking if medium duration of illness	Proportion of cases of AG with a duration of illness of 3-5 days that consulted for their illness, estimated from a national telephone survey conducted in 2009-2010 (*1*)	log normal	meanlog: -0.865, sdlog: 0.142
Proportion of cases with a medium duration of illness	Proportion of *Salmonella* spp. cases with a duration of illness of 3-5 days used as a proxy	Beta	α: 186.85, β:210.71
Medical care seeking if short duration of illness	Proportion of cases of AG with a duration of illness less than 3 days that consulted for their illness, estimated from a national telephone survey conducted in 2009-2010 (*1*)	log normal	meanlog: -1.708, sdlog: 0.216
Proportion of cases with a short duration of illness	Proportion of *Salmonella* spp. cases with a duration of illness less than 5 days used as a proxy	Beta	α: 383.96, β: 9,215.04
Reported hospitalizations	Annual number of patients hospitalized with an ICD-10 code of shigellosis (A03.0 – A03.9) reported in the French hospital information system (2008-2013)	log normal	meanlog: 5.759, sdlog: 0.117
Reported deaths	Annual number of patients hospitalized with an ICD-10 code of shigellosis (A03.0 – A03.9) reported in the French hospital information system (2008-2013) with "death" coded as the mode of discharge	log normal	meanlog: 0, sdlog: 1
Laboratory testing: hospitalizations/deaths	Proportion of stool samples tested for *Shigella* spp. in hospital laboratories assumed to be 100%	Fixed	100%
Test sensitivity: hospitalizations/deaths	Sensitivity of stool culture for *Shigella* spp. assumed to be between 90 and 100%	Beta	α 71.25, β: 3.75
Proportion domestic	Proportion of cases with no travel history reported by the national reference center between 2008 and 2013 (71 – 78%)	Beta	α: 461.398, β: 157.928
Proportion foodborne	Estimated to be between 23 and 40% based on US estimates published in 2011 (*2*)	Beta	α: 37.315, β: 81.1455
Staphylococcus aureus			
Reported illnesses	Annual number of reported foodborne outbreak cases reported to the French national public health agency between 2008 and 2013	log normal	meanlog: 5.727, sdlog: 0.619
Underreporting	*Salmonella* spp. data used as a proxy (2008-2013) : annual number of reported foodborne outbreak cases (min 509 - max 1 066) divided by the annual number of food-related laboratory confirmed cases min 13 872 – max 15 074)	Beta	α: 25.11, β: 427.49
Test sensitivity	Sensitivity of stool culture for *Salmonella* spp. used as a proxy	Beta	α: 71.25, β: 3.75
Laboratory testing	The proportion of stool samples tested for *Salmonella* spp. used as a proxy	Beta	α: 71.25, β: 3.75
Specimen submission	Proportion of stool samples prescribed for *Salmonella* spp. used as a proxy	Beta	α: 21.36, β: 108.08
Medical care seeking if long duration of illness	Proportion of cases of AG with a duration of illness longer than 5 days that consulted for their illness, estimated from a national telephone survey conducted in 2009-2010 (*1*)	log normal	meanlog: -0.346, sdlog: 0.141
Proportion of cases with a long duration of illness	Proportion of *Salmonella* spp. cases with a duration of illness longer than 5 days used as a proxy	Beta	α: 195.43, β: 203.41
Medical care seeking if medium duration of illness	Proportion of cases of AG with a duration of illness of 3-5 days that consulted for their illness, estimated from a national telephone survey conducted in 2009-2010 (*1*)	log normal	meanlog: -0.865, sdlog: 0.142
Proportion of cases with a medium duration of illness	Proportion of *Salmonella* spp. cases with a duration of illness of 3-5 days used as a proxy	Beta	α: 186.85, 210.71
Medical care seeking if short duration of illness	Proportion of cases of AG with a duration of illness of less than 3 days that consulted for their illness, estimated from a national telephone survey conducted in 2009-2010 (*1*)	log normal	meanlog: -1.708, sdlog: 0.216
Proportion of cases with a short duration of illness	Proportion of *Salmonella* spp. cases with a duration of illness of less than 3 days used as a proxy	Beta	α: 383.96, β: 9,215.04
Proportion hospitalized	Proportion estimated for acute gastroenteritis used as a proxy (*cf*. table acute gastroenteritis in this appendix)	Beta	α: 229.15.11, β: 34,255.89
Proportion of hospitalized patients who died	Proportion estimated for norovirus used as a proxy (*cf*. table norovirus in this appendix)	Beta	α: 63.84, β: 26,537.82
Proportion foodborne	Only foodborne outbreak cases reported in France. Proportion estimated to be 100%, similar to US estimates published in 2011 (*2*)	Fixed	100%
Taenia saginata			
Illnesses	Annual number of persons with a reimbursement of Niclosamide treatment between 2011 and 2013	log normal	meanlog: 10.405, sdlog: 0.0686
Reported hospitalizations	Annual number of cases hospitalized with an ICD-10 code related to taeniais (B68.1 - B68.9) reported in the French hospital information system (2008-2013)	log normal	meanlog: 4.920, sdlog: 0.0657
Reported deaths	Annual number of cases hospitalized with an ICD-10 code related to taeniais (B68.1 - B68.9) reported in the French hospital information system (2008-2013) with "death" coded as the mode of discharge	log normal	meanlog: 1.386, sdlog: 0.5
Proportion foodborne	Only foodborne transmission documented in France, proportion estimated to be 100%	Fixed	100%
Toxoplasma gondii (acquired and congenital)†			
Illnesses	Annual number of infections of toxoplasmosis estimated by modelling age-and time specific seroprevalence data (2010-2011)‡	log normal	meanlog: 11.983, sdlog: 0.018
Proportion symptomatic	Proportion of symptomatic illness estimated between 10 and 20% (*22*)	Beta	α: 30.45, β: 172.55
Reported hospitalizations	Annual number of cases hospitalized with a specific ICD-10 code (B58.0 - B58.9) reported in the French hospital information system (2008-2013)	log normal	meanlog: 7.092, sdlog: 0.0549
Reported deaths	Annual number of cases hospitalized with a specific ICD-10 code (B58.0 - B58.9) reported in the French hospital information system (2008-2013) with "death" coded as the mode of discharge	log normal	meanlog: 3.807, sdlog: 0.266

Model input	Data source(s)	Distribution	Data for model input
Proportion foodborne	Estimated to be between 40 and 60% based on US estimates published in 2011 (*2*)	Beta	α: 49.5, β: 49.5
Yersinia spp.			
Reported illnesses	Annual number of stool cultures reimbursed in the national health insurance database between 2010 and 2012	log normal	meanlog: 13.349, sdlog: 0.036
Underreporting	The frequency of *Yersinia* spp. isolation from stool cultures estimated from a national laboratory survey carried out in 2004-2005 (*23*)	Beta	α: 19.745, β: 5,703.455
Test sensitivity	Sensitivity of stool culture for *Yersinia* spp., assumed to be between 80 and 100%	Beta	α 31.5, β: 3.5
Laboratory testing	Proportion of stool samples tested for *Campylobacter* spp. used as a proxy	Beta	α: 81.121, β: 44.648
Specimen submission	Proportion of stool samples prescribed for *Campylobacter* spp. used as a proxy	Beta	α: 17.792, β: 83.875
Medical care seeking if long duration of illness	Proportion of cases of AG with a duration of illness longer than 5 days that consulted for their illness, estimated from a national telephone survey conducted in 2009-2010 (*1*)	log normal	meanlog: -0.346, sdlog: 0.141
Proportion of cases with a long duration of illness	Proportion *Campylobacter* spp. cases with a duration of illness longer than 5 days, used as a proxy	Beta	α: 118.63, β: 48.45
Medical care seeking if medium duration of illness	Proportion of cases of AG with a duration of illness of 3-5 days that consulted for their illness, estimated from a national telephone survey conducted in 2009-2010 (*1*)	log normal	meanlog: -0.865, sdlog: 0.142
Proportion of cases with a medium duration of illness	Proportion *Campylobacter* spp. cases with a duration of illness of 3-5 days used as a proxy	Beta	Α: 236.25, β: 638.75
Medical care seeking if short duration of illness	Proportion of cases of AG with a duration of illness less than 3 days that consulted for their illness, estimated from a national telephone survey conducted in 2009-2010 (*1*)	log normal	meanlog: -1.708, sdlog: 0.216
Proportion of cases with a short duration of illness	Proportion *Campylobacter* spp. cases with a duration of illness less than 3 days used as a proxy	Beta	α: 391.98, β: 19,207.02
Reported hospitalizations	Annual number of patients hospitalized with an ICD-10 code of yersiniosis (A04.6) reported in the French hospital information system (2008-2013)	log normal	meanlog: 5.371, sdlog: 0.093
Reported deaths	Annual number of patients hospitalized with an ICD-10 code of yersiniosis (A04.6) reported in the French hospital information system (2008-2013) with “death” coded as the mode of discharge	log normal	meanlog: 2.303, sdlog: 1.4
Laboratory testing: hospitalizations/deaths	Proportion of stool samples tested for *Campylobacter* spp. tested in hospital laboratories used as a proxy	Beta	α: 46.326, β: 16.277
Test sensitivity: hospitalizations/deaths	Sensitivity of stool culture for *Yersinia* spp. assumed between 80% and 100%	Beta	α 31.5, β: 3.5
Proportion pathogenic	Proportion of pathogenic strains of *Yersinia* spp. reported by the national reference center between 2008 and 2013 (66-71%)	Beta	α 945.273, β: 434.688
Proportion foodborne	Estimated to be between 80 and 100% based on US estimates published in 2011 (*2*)	Beta	α 31.5, β: 3.5

*We used seroprevalence data from 3 different seroprevalence surveys in France to derive age-specific seroprevalence estimates for hepatitis A virus (HAV). We used surveillance data (mandatory notification) from domestic symptomatic cases of HAV reported to the French national public health agency between 2008 and 2013 to produce age-specific incidence estimates. The incidence of HAV infection in France was then estimated using a catalytic model as published by Armstrong et al. in the United States (*10*) and Pham et al. in Canada (*11*). Using this model the sensibility of our surveillance system was estimated to be 6.92% (95% confidence interval 6.55–7.3%).
†The estimates derived from modelling the seroprevalence data and from the French hospital information system comprise both acquired and congenital toxoplasmosis. A very small proportion of these cases are congenital toxoplasmosis: in France 256 cases of congenital toxoplasmosis were annually reported by the national laboratory surveillance system (2008-2013), of which 90% were asymptomatic (*24*)
‡We estimated the incidence of *T. gondii* infection using age-and time specific seroprevalence data from 6 different seroprevalence surveys in France, following a catalytic epidemic parametric model proposed by Ades and Nokes (*25*). This model-based approach has recently been used and adapted in France to estimate the incidence and prevalence of *T. gondii* infections among women in France (*26*). We applied this model to the general population (unpublished data).

Technical Appendix Table 2. Estimates (5th, 50th, 95th percentiles of the output distribution) of the annual number of illnesses, hospitalizations and deaths caused by foodborne pathogens, France, 2008–2013*

	All modes of transmission†			Proportion foodborne	Foodborne transmission†		
Pathogen	5%	50%	95%	transmission, %	5%	50%	95%
Bacillus cereus				100			
Illnesses	32,841	69,468	164,316		32,841	69,468	164,316
Hospitalizations	216	457	1,080		216	457	1,080
Deaths	0.5	1.1	2.6		0.5	1.1	2.6
Campylobacter spp.				73–86			
Illnesses	272,669	492,705	1,078,543		215,216	392,177	862,747
Hospitalizations	5,138	6,943	9,510		4,039	5,524	7,595
Deaths	33	52	82		26	41	65
Clostridium botulinum				100			
Illnesses	11	21	41		11	21	41
Hospitalizations	10	19	37		10	19	37
Deaths	0.02	0.3	3.7		0.02	0.3	3.7
Clostridium perfringens				100			
Illnesses	47,922	119,632	332,244		47,922	119,632	332,244
Hospitalizations	317	811	2,238		317	811	2,238
Deaths	1	2	6		1	2	6
STEC				59–87			
Illnesses	11,523	24,710	52,295		8,206	17,927	38,668

Pathogen	All modes of transmission† 5%	50%	95%	Proportion foodborne transmission, %	Foodborne transmission† 5%	50%	95%
Hospitalizations	199	514	1,259		143	372	928
Deaths	2	6	16		2	4	12
Listeria monocytogenes				100			
Illnesses	328	402	497		328	402	497
Hospitalizations	258	310	375		258	310	375
Deaths	47	65	90		46	65	90
Salmonella spp.				91–95			
Illnesses	108,805	198,047	410,817		102,041	183,002	387,599
Hospitalizations	3,927	4,415	4,983		3,644	4,106	4,632
Deaths	62	72	84		57	67	78
Shigella spp.				23–40			
Illnesses	6,206	11,082	23,143		1,837	3,449	7,555
Hospitalizations	204	248	305		56	78	104
Deaths	0.2	0.8	4		0.05	0.3	1.3
Staphylococcus aureus				100			
Illnesses	21,058	73,021	271,056		21,058	73,021	271,056
Hospitalizations	141	486	1,827		141	486	1,827
Deaths	0.3	1.2	4.3		0.3	1.2	4.3
Yersinia spp.				80–100			
Illnesses	12,175	23,674	54,388		10,799	21,330	49,477
Hospitalizations	180	222	278		158	200	255
Deaths	1	10	108		1	9	96
Hepatitis A virus				16			
Illnesses	12,658	16,416	21,384		2,025	2,627	3,421
Hospitalizations	1,130	1,567	2,162		181	251	346
Deaths	24	30	37		4	5	6
Hepatitis E virus				75–100			
Illnesses	46,032	68,007	101,279		39,388	59,320	88,967
Hospitalizations	540	546	553		413	482	524
Deaths	18	20	22		15	18	20
Norovirus				12–16			
Illnesses	2,971,892	3,706,693	4,579,554		402,816	517,593	656,921
Hospitalizations	19,271	24,659	31,161		2,610	3,447	4475
Deaths	43	59	80		6	8	12
Taenia saginata				100			
Illnesses	29,487	33,006	36,946		29,487	33,006	36,946
Hospitalizations	123	137	153		123	137	153
Deaths	2	4	9		2	4	9
Toxoplasma gondii‡				40–60			
Illnesses	17,567	23,786	30,824		8,401	11,785	16,133
Hospitalizations	1,097	1,202	1,315		493	601	719
Deaths	29	45	69		14	22	35
Subtotal bacteria							
Illnesses	772,257	1,012,762	1,826,612		673,683	880,429	1,594,203
Hospitalizations	12,574	14,425	18,196		10,791	12,363	15,818
Deaths	182	210	315		166	191	288
Subtotal viruses							
Illnesses	3,073,457	3,791,116	4,682,930		463,131	579,540	723,256
Hospitalizations	21,320	26,772	33,444		3,344	4,180	5,189
Deaths	91	109	132		27	31	35
Subtotal parasites							
Illnesses	49,658	56,792	64,942		38,880	44,791	50,640
Hospitalizations	1,234	1,339	1,455		630	738	858
Deaths	33	49	73		18	31	41
Total							
Illnesses	4,175,457	4,860,670	6,155,454		1,280,977	1,504,760	2,233,664
Hospitalizations	37,242	42,536	50,526		15,520	17,281	20,785
Deaths	335	368	486		223	248	350

*STEC, Shiga-toxin–producing *Escherichia coli*.
†5%: 5th percentile; 50%: median; 95%: 95 percentile of the output distribution of estimates for illnesses, hospitalizations and deaths.
‡We considered both acquired and congenital toxoplasmosis.

References

1. Van Cauteren D, De Valk H, Vaux S, Le Strat Y, Vaillant V. Burden of acute gastroenteritis and healthcare-seeking behaviour in France: a population-based study. Epidemiol Infect. 2012;140:697–705. PubMed http://dx.doi.org/10.1017/S0950268811000999

2. Scallan E, Hoekstra RM, Angulo FJ, Tauxe RV, Widdowson MA, Roy SL, et al. Foodborne illness acquired in the United States—major pathogens. Emerg Infect Dis. 2011;17:7–15. PubMed http://dx.doi.org/10.3201/eid1701.P11101

3. Van Cauteren D, De Valk H, Sommen C, King LA, Jourdan-Da Silva N, Weill FX, et al. Community incidence of campylobacteriosis and nontyphoidal salmonellosis, France, 2008–2013. Foodborne Pathog Dis. 2015;12:664–9. PubMed http://dx.doi.org/10.1089/fpd.2015.1964

4. Bessède E, Delcamp A, Sifré E, Buissonnière A, Mégraud F. New methods for detection of campylobacters in stool samples in comparison to culture. J Clin Microbiol. 2011;49:941–4. PubMed http://dx.doi.org/10.1128/JCM.01489-10

5. Van Cauteren D, Turbelin C, Fonteneau L, Hanslik T, De Valk H, Blanchon T. Physician practices in requesting stool samples for patients with acute gastroenteritis, France, August 2013–July 2014. Epidemiol Infect. 2015;143:2532–8. PubMed http://dx.doi.org/10.1017/S0950268814003884

6. Gallay A, Bousquet V, Siret V, Prouzet-Mauléon V, Valk H, Vaillant V, et al. Risk factors for acquiring sporadic *Campylobacter* infection in France: results from a national case-control study. J Infect Dis. 2008;197:1477–84. PubMed http://dx.doi.org/10.1086/587644

7. Kuehne A, Bouwknegt M, Havelaar A, Gilsdorf A, Hoyer P, Stark K, et al.; and the HUS active surveillance network Germany. Estimating true incidence of O157 and non-O157 Shiga toxin–producing *Escherichia coli* illness in Germany based on notification data of haemolytic uraemic syndrome. Epidemiol Infect. 2016;144:3305–15. PubMed http://dx.doi.org/10.1017/S0950268816001436

8. Havelaar AH, Haagsma JA, Mangen MJ, Kemmeren JM, Verhoef LP, Vijgen SM, et al. Disease burden of foodborne pathogens in the Netherlands, 2009. Int J Food Microbiol. 2012;156:231–8. PubMed http://dx.doi.org/10.1016/j.ijfoodmicro.2012.03.029

9. Majowicz SE, Scallan E, Jones-Bitton A, Sargeant JM, Stapleton J, Angulo FJ, et al. Global incidence of human Shiga toxin–producing *Escherichia coli* infections and deaths: a systematic review and

knowledge synthesis. Foodborne Pathog Dis. 2014;11:447–55. PubMed http://dx.doi.org/10.1089/fpd.2013.1704

10. Armstrong GL, Bell BP. Hepatitis A virus infections in the United States: model-based estimates and implications for childhood immunization. Pediatrics. 2002;109:839–45. PubMed http://dx.doi.org/10.1542/peds.109.5.839

11. Pham B, Chen MH, Tricco AC, Anonychuk A, Krahn M, Bauch CT. Use of a catalytic model to estimate hepatitis A incidence in a low-endemicity country: implications for modeling immunization policies. Med Decis Making. 2012;32:167–75. PubMed http://dx.doi.org/10.1177/0272989X11398489

12. Petrignani M, Verhoef L, Vennema H, van Hunen R, Baas D, van Steenbergen JE, et al. Underdiagnosis of foodborne hepatitis A, The Netherlands, 2008–2010(1.). Emerg Infect Dis. 2014;20:596–602. PubMed http://dx.doi.org/10.3201/eid2004.130753

13. Pillonel J, Gallian P, Sommen C, Couturier E, Piquet Y, Djoudi R, et al.; Comité de Pilotage de la Surveillance Épidémiologique des Donneurs de Sang. Assessment of a transfusion emergent risk: the case of HEV [in French]. Transfus Clin Biol. 2014;21:162–6. PubMed http://dx.doi.org/10.1016/j.tracli.2014.07.004

<jrn>14. Pillonel J, Legrand D, Sommen C, Laperche S. Epidemiological surveillance of blood donors and residual risk of transmission of HIV, HTLV, HCV and HBV by transfusion in France between 2008 and 2010 [in French]. Bull Epidemiol Hebd (Paris). 2012;39-40:438–42.</jrn>

15. Said B, Ijaz S, Kafatos G, Booth L, Thomas HL, Walsh A, et al.; Hepatitis E Incident Investigation Team. Hepatitis E outbreak on cruise ship. Emerg Infect Dis. 2009;15:1738–44. PubMed http://dx.doi.org/10.3201/eid1511.091094

16. Guillois Y, Abravanel F, Miura T, Pavio N, Vaillant V, Lhomme S, et al. High proportion of asymptomatic infections in an outbreak of hepatitis E associated with a spit-roasted piglet, France, 2013. Clin Infect Dis. 2016;62:351–7. PubMed http://dx.doi.org/10.1093/cid/civ862

17. Tam CC, O’Brien SJ, Tompkins DS, Bolton FJ, Berry L, Dodds J, et al.; IID2 Study Executive Committee. Changes in causes of acute gastroenteritis in the United Kingdom over 15 years: microbiologic findings from 2 prospective, population-based studies of infectious intestinal disease. Clin Infect Dis. 2012;54:1275–86. PubMed http://dx.doi.org/10.1093/cid/cis028

18. Tam CC, Rodrigues LC, Viviani L, Dodds JP, Evans MR, Hunter PR, et al.; IID2 Study Executive Committee. Longitudinal study of infectious intestinal disease in the UK (IID2 study): incidence

in the community and presenting to general practice. Gut. 2012;61:69–77. PubMed http://dx.doi.org/10.1136/gut.2011.238386

19. Ahmed SM, Hall AJ, Robinson AE, Verhoef L, Premkumar P, Parashar UD, et al. Global prevalence of norovirus in cases of gastroenteritis: a systematic review and meta-analysis. Lancet Infect Dis. 2014;14:725–30. PubMed http://dx.doi.org/10.1016/S1473-3099(14)70767-4

20. Verhoef L, Hewitt J, Barclay L, Ahmed SM, Lake R, Hall AJ, et al. Norovirus genotype profiles associated with foodborne transmission, 1999–2012. Emerg Infect Dis. 2015;21:592–9. PubMed http://dx.doi.org/10.3201/eid2104.141073

21. Carrillo-Santisteve P, Jourdan-Da Silva N, Weill FX, Le Hello S, Fromage M, de Valk H. Completeness of reporting to the voluntary *Salmonella* surveillance system in France, 2008. Stockholm: ESCAIDE. 2010.

22. Montoya J, Boothroyd J, Kovacs J. *Toxoplasma gondii*. In: Mandell GL, Bennett JE, Dolin R, editors. Mandell, Douglas, and Bennett's principles and practice of infectious diseases. 7th ed. London: Churchill Livingstone; 2010. p. 3496–526.

23. Savin CLA, Laurent E, Carniel E, Vaillant V. National survey on diagnosis of enteropathogenic *Yersinia* infections in metropolitan France, 2003 [in French]. Bull Epidemiol Hebd (Paris). 2010;29:307–11.

24. Villena I, Ancelle T, Delmas C, Garcia P, Brezin AP, Thulliez P, et al.; Toxosurv network and National Reference Centre for Toxoplasmosis. Congenital toxoplasmosis in France in 2007: first results from a national surveillance system. Euro Surveill. 2010;15:19600. PubMed

25. Ades AE, Nokes DJ. Modeling age- and time-specific incidence from seroprevalence: toxoplasmosis. Am J Epidemiol. 1993;137:1022–34. PubMed http://dx.doi.org/10.1093/oxfordjournals.aje.a116758

26. Nogareda F, Le Strat Y, Villena I, De Valk H, Goulet V. Incidence and prevalence of *Toxoplasma gondii* infection in women in France, 1980–2020: model-based estimation. Epidemiol Infect. 2014;142:1661–70. PubMed http://dx.doi.org/10.1017/S0950268813002756
